# Aniridia-related keratopathy relevant cell signaling pathways in human fetal corneas

**DOI:** 10.1007/s00418-022-02099-9

**Published:** 2022-05-12

**Authors:** André Vicente, Marta Sloniecka, Jing-Xia Liu, Berit Byström, Fátima Pedrosa Domellöf

**Affiliations:** 1 Department of Clinical Sciences, Ophthalmology, Umeå University, 901 85 Umeå, Sweden; 2 Department of Integrative Medical Biology, Section for Anatomy, Umeå University, Umeå, Sweden

**Keywords:** Aniridia, Fetal cornea, Adult cornea, Sonic hedgehog, Notch, mTOR, Wnt

## Abstract

We aimed to study aniridia-related keratopathy (ARK) relevant cell signaling pathways [Notch1, Wnt/β-catenin, Sonic hedgehog (SHH) and mTOR] in normal human fetal corneas compared with normal human adult corneas and ARK corneas. We found that fetal corneas at 20 weeks of gestation (wg) and normal adult corneas showed similar staining patterns for Notch1; however 10–11 wg fetal corneas showed increased presence of Notch1. Numb and Dlk1 had an enhanced presence in the fetal corneas compared with the adult corneas. Fetal corneas showed stronger immunolabeling with antibodies against β-catenin, Wnt5a, Wnt7a, Gli1, Hes1, p-rpS6, and mTOR when compared with the adult corneas. Gene expression of Notch1, Wnt5A, Wnt7A, β-catenin, Hes1, mTOR, and rps6 was higher in the 9–12 wg fetal corneas compared with adult corneas. The cell signaling pathway differences found between human fetal and adult corneas were similar to those previously found in ARK corneas with the exception of Notch1. Analogous profiles of cell signaling pathway activation between human fetal corneas and ARK corneas suggests that there is a less differentiated host milieu in ARK.

**Supplementary Information:**

The online version contains supplementary material available at 10.1007/s00418-022-02099-9.

## Introduction

Aniridia is a congenital autosomal dominant disease caused by haploinsufficiency of the *PAX6* gene transcription factor (Lim et al. 2017). It includes, among other clinical features, aniridia-related keratopathy (ARK), which classically occurs after childhood and recurs even if the patients undergo corneal transplantation (Bausili et al. 2016; Vicente et al. 2018a). This chronic progressive keratopathy is characterized by a multitude of defects, including disturbed corneal limbal cell differentiation, fragile epithelial cells, compromised epithelial cell adhesion, and a chronic wound-healing state with compromised barrier function, which result in epithelial erosions, corneal conjunctivalization, and vascular pannus with significant impact on vision (Ou et al. 2008; Ramaesh et al. 2005; Latta et al. 2018). We have recently reported altered cell signaling pathways in ARK, with decreased detection of the Notch1 cell signaling pathway and enhanced activation of the Sonic hedgehog (SHH), mTOR, and Wnt/β-catenin cell signaling pathways not only in the epithelium but also in the subepithelial pannus (Vicente et al. 2018b).

The development of the cornea includes two waves of neural crest cells, and the primitive corneal epithelium appears around day 33. The epithelium is then two-layered and already has a basement membrane (Remington and McGill 1998). At 7 weeks of gestation (wg), mesenchymal cells migrate to form the corneal stroma and endothelium, and at 7.5 wg the keratoblasts, which will later differentiate into keratocytes, are arranged in four to five incomplete layers with few collagen fibrils. The corneal epithelium continues to evolve into a stratified squamous epithelium (Herwig et al. 2013), and by 11–12 wg all corneal layers are formed, with the exception of the Bowman’s layer (Remington and McGill 1998). The epithelium has then two to three cell layers, and the stroma has 25–30 layers of keratoblasts and is separated from the endothelium by an irregular Descemet’s membrane (Cook CS 1994). The Bowman’s layer is formed around 16–17 wg (Wulle and Richter 1978), and by then the keratoblasts are distributed in a disorganized pattern in the anterior stroma (Cook CS 1994). At 7 months of gestation, the cornea already has an adult structure but in the anterior stroma there are more keratoblasts and the collagen lamellae are still more randomly oriented (Remington and McGill 1998). The corneal structures generally develop adult features after the first 2 years of life (Herwig et al. 2013; Bystrom et al. 2006).

Notch1, Wnt/β-catenin, SHH, and mTOR cell signaling pathways are essential for eye development, regulate both cell proliferation and homeostasis in mammals, and are altered in ARK corneas (Penton et al. 2012; Brennan and Giles 2013; Hagglund et al. 2017; Wang et al. 2018; Vicente et al. 2018b). The Notch1 signaling pathway coordinates corneal epithelial repair, vertical migration, and regulation of basal corneal epithelial cells. In the central corneal epithelium, these processes depend on transient amplifying cells (TAC), which have high migratory and proliferative capacity (Zhou et al. 2006). TAC are derived from limbal epithelial stem cells during normal corneal epithelial homeostasis and wound healing processes and migrate centripetally in the basal layer to maintain the corneal epithelium (Lehrer et al. 1998). Dlk1, a transmembrane protein that functions as negative regulator of Notch1, ensures that cells are kept in progenitor mode, which is essential in developmental processes (Falix et al. 2012). We have previously shown that Dlk1 activation is increased in ARK corneal epithelial cells and stroma (Vicente et al. 2018b). Numb, another important negative regulator of Notch1, is also upregulated in ARK corneas (Vicente et al. 2018b).

Determination of limbal stem cell fate is regulated by the Notch1 and Wnt/β-catenin signaling pathways, which are essential during normal tissue development and regeneration (Wang et al. 2018; Tsai et al. 2014). The extracellular ligands Wnt5a and Wnt7a stimulate the Wnt/β-catenin signaling cascade, which leads to proliferation of corneal limbal stem cells (Nakatsu et al. 2011), and is increased in ARK corneas (Vicente et al. 2018b); β-catenin is important for the regulation of epithelial differentiation and stratification (Li et al. 2015).

The SHH pathway regulates maintenance of cell polarity, cell differentiation, and proliferation and leads to activation of the glioma-associated oncogene homolog (Gli1) transcription factor that controls cell growth and survival (Taipale and Beachy 2001). This pathway is upregulated in case of corneal epithelium debridement (Saika et al. 2004) and in ARK corneas (Vicente et al. 2018b). It shares a downstream effector, Hes1, with the Notch1 signaling pathway, which is essential for the regulation of corneal epithelial stem cells and mammalian eye development (Lee et al. 2005). The serine/threonine kinase mammalian target of rapamycin (mTOR) signaling pathway regulates cell growth and proliferation. Activation of the mTOR signaling pathway leads to phosphorylation of the ribosomal protein S6 (rpS6) (Dowling et al. 2010). mTOR signaling is upregulated in ARK corneas (Vicente et al. 2018b), and premature upregulation of mTORC1 during development in mice causes aniridia and anterior segment dysgenesis (Braune and Lendahl 2016; Wang et al. 2018; Hagglund et al. 2017).

How these signaling pathways contribute to development of the human fetal cornea is currently poorly understood. Given that many tissues recapitulate patterns of fetal maturation during regeneration, in the present study we employed immunostaining in conjunction with fluorescence microscopy and qPCR to comparatively analyze potential divergence of the important developmental signaling pathways Notch1, Wnt/β-catenin, SHH, and mTOR between human fetal, normal adult, and ARK corneas.

## Methods

### Corneal samples

Eyes from eight human fetuses were collected, with ethical approval, after legal interruptions of pregnancy at 9 wg (*n* = 2), 10–11 wg (*n* = 2), 12 wg (*n* = 2), and 20 wg (*n* = 2). Gestational age was calculated on the basis of the first day of the last menstrual period and confirmed with ultrasound. Five adult healthy corneas were collected from four male (74, 76, 82, and 83 years old) and one female (37 years old) donors. These normal corneas were obtained from deceased individuals who, when alive, chose to donate their eyes post mortem for transplantation and research purposes, according to Swedish law. The Regional Ethical Review Board in Umeå has determined the use of the post-mortem donated, anonymized tissue for study purposes to be exempt from the requirement for approval. In addition, the corneas donated by three patients with aniridia undergoing surgery were also included for reference. More information on these ARK corneas and how they were processed can be found in the study of Vicente et al. (2018b), as cases A–C. The study followed the principles of the Declaration of Helsinki.

The two human adult corneas (from 74- to 82-year-old males) used for immunochemistry were first routinely fixed in 10% buffered formalin for 24 h at room temperature and embedded into paraffin wax. SuperFrost Plus slides were used to collect serial sections, 5 μm thick, which were dried in a vertical position overnight at 60 °C and then placed at +4 °C in a slide box, as previously described (Vicente et al. 2018b). The remaining three adult corneas and the fetal samples 10–11 and 20 wg were mounted on cardboard with embedding medium (OCT cryomount, HistoLab Products AB, Gothenburg, Sweden), quickly frozen in propane cooled with liquid nitrogen and stored at −80 °C. For immunohistochemistry 5-μm-thick serial sections were cut at −23 °C using a Leica CM3050 cryostat (Leica Microsystems, Wetzlar, Germany) and collected on SuperFrost Plus slides (Thermo Fisher Scientific, Rockford, IL, USA). For laser microdissections, 10-µm-thick serial sections of freshly frozen adult corneas, as well as 9 wg, 10–11 wg, and 12 wg fetal corneas were cut in RNase-free conditions at −23 °C using a Leica CM3050 cryostat, and collected on NF1.0 PEN membrane slides (Zeiss, Jena, Germany). Sections were fixed in ice-cold 70% ethanol for 1 min and transferred to a −80 °C freezer until further processing.

### Immunohistochemistry

The sections of the human fetal corneas were first brought to room temperature, left to dry (20 min), and then rinsed three times (5 min) in 001 M phosphate-buffered saline (PBS). Sections from adult control healthy corneas and ARK corneas were dewaxed with Tissue-Clear (1466, Sakura Finetek Europe, Alphen aan den Rijn, Netherlands) and rehydrated. Antigen retrieval was performed with a water bath at 95 °C (30 min) in prewarmed citrate buffer (10 mM citric acid, 0.05% Tween 20, pH 6.0) or Tris–EDTA buffer (10 mM Tris base, 1 mM EDTA, 0.05% Tween 20 pH 9.0). The sections were put at room temperature (20 min) to cool down, washed in running water (5 min), and then submerged in 0.01 M PBS, three times (5 min).

To block unspecific binding, all corneal sections were incubated with 5% normal goat or donkey serum at room temperature (15 min). Thereafter, the sections were incubated with either monoclonal or polyclonal primary antibodies (Abs) specific against components of the Notch1 (Notch1, Dlk1, Numb), Wnt/β-catenin (Wnt5a, Wnt7a, β-catenin), Sonic Hedgehog (Gli1, Hes1), and mTOR (mTOR1, p-rpS6) signaling pathways (Table 1), at +4 °C overnight. The sections were subsequently incubated with the adequate secondary antibody (Table 2) at 37 °C for 30 min, submerged in 0.01 M PBS three times (5 min), and then mounted with Vectashield mounting medium with 4′,6-diamidino-2-phenylindole (DAPI; H-1000 and H-1200; Vector Laboratories, Burlingame, USA). Potential unspecific binding of secondary antibodies was evaluated by omission of the primary antibody in sections that were otherwise processed similarly.

**Table 1:** Primary antibodies

Antigen	Antibody	Concentration	Source
Dlk1	Ab21682	1:1000	Abcam, Cambridge, UK
Notch1	Ab52627	1:300	Abcam, Cambridge, UK
Numb	Ab14140	1:250	Abcam, Cambridge, UK
Wnt5a	Ab32572	1:100	Abcam, Cambridge, UK
Wnt7a	Bs-6645R	1:100	Bioss, Woburn, MA, USA
β-catenin	Ab32572	1:100	Abcam, Cambridge, UK
Hes1	GTX 108,356	1:500	Genetex, Irvine, CA, USA
Gli1	Ab 92,611	1:800	Abcam, Cambridge, UK
mTOR	(7C10) 2983	1:500	Cell Signaling, Danvers, MA, USA
p-rpS6	GTX 60,800	1:800	Genetex, Irvine, CA, USA

**Table 2:** Secondary antibodies (IgG)

Antibody	Catalog number	Concentration	Source
Donkey anti-mouse DyLight 488	715–485-150	1:100	Jackson ImmunoResearch Europe Ltd, Suffolk, UK
Goat anti-mouse Alexa 488	A21121	1:300	Molecular Probes, Life Technologies, Darmstadt, Germany
Donkey anti-rabbit FITC	715–095-152	1:50	Jackson ImmunoResearch Europe Ltd, Suffolk, UK
Goat anti-rabbit Alexa 488	A11034	1:300	Molecular Probes, Life Technologies, Darmstadt, Germany

We have previously worked with both freshly frozen and formalin-fixed corneal specimens, using the antibodies included in the present study. We have previously ascertained that the staining pattern was similar irrespective of the tissue being freshly frozen or paraffin embedded (Vicente et al. 2018a; Bystrom et al. 2009, 2007, 2006).

### Image acquisition

The sections were photographed under a Leica DM 6000 B microscope (Leica Microsystems, Wetzlar, Germany), equipped with a 1.4-megapixel Leica DFC360 FX digital camera (Leica Microsystems, Wetzlar, Germany) using Leica Application Suite X (LAS X) software version 3.6.0.20104 (Leica Microsystems CMS, GmbH, Wetzlar, Germany), with the following fluorescence filters: A4/DAPI-400-blue, L5/FITC-488-green, and TXR-594-red. Image processing was performed using Adobe Photoshop CS6 software (Adobe Systems, San Jose, CA, USA). Both the immunostaining procedures and image acquisition with fluorescence microscopy were performed using identical settings for all samples. Therefore, relative comparisons between different samples treated with the same antibody could be performed.

### Laser microdissections

Before laser microdissection (LMD) was performed, the NF1.0 PEN membrane slides with 9 wg, 10–11 wg, and 12 wg fetal corneas and adult control corneas were transferred from −80 °C directly to ice-cold 70% ethanol for 2 min, washed in nuclease-free water to remove residual OCT, and then dehydrated in 70%, 96%, and 100% ethanol for 20 s each. One additional wash in 100% ethanol was performed for 1 min. Afterwards, the slides were left to dry inside a fume hood for 10 min and transferred to a desiccator. LMD was performed using a PALM MicroBeam microscope (Carl Zeiss Microscopy, Jena, Germany). The central part of the corneas (stroma and epithelium) was carefully dissected, pooled (8 sections from each fetal cornea, and 16 sections from each adult cornea), lysed in lysis buffer, and used for gene expression analysis.

### RT-qPCR

mRNA was extracted from the dissected corneas using an RNA extraction kit (Qiagen, Venlo, Netherlands) according to the manufacturer’s instructions. Next, 35 ng of RNA was reverse transcribed into cDNA using a high-capacity cDNA reverse transcription kit (Thermo Fisher). Notch1 (*NOTCH1*; Hs01062014_m1), Dlk1 (*DLK1*; Hs00171584_m1), Numb (*NUMB*; Hs01105433_m1), Wnt5A (*WNT5A*; Hs00998537_m1), Wnt7A (*WNT7A*; Hs01114990_m1), β-catenin (*CTNNB1*; Hs00355045_m1), Hes1 (*HES1*; Hs00172878_m1), mTOR (*mTOR*, Hs00234508_m1), and rpS6 (*RPS6*; Hs01058685_g1) probes were used to determine gene expression (Thermo Fisher). Samples were run in quadruplicate in two separate experiments in ViiA 7 Real-Time PCR System (Thermo Fisher). β-Actin and 18S probes served as endogenous controls (Thermo Fisher). Analysis was performed with ViiA 7 Software (Thermo Fisher). Because of the small sample size, the fold changes in gene expression of the fetal corneas at 9 wg, 10–11 wg, and 12 wg were pooled together and compared with those of the adult corneas. Additionally, Supplemental Fig. 1 shows fold change gene expression in the fetal corneas at 9 wg and 12 wg separately as compared with adult corneas.

### Statistical analysis

Data are presented as mean ± standard deviation (SD). Statistical analyses were performed with nonparametric Mann–Whitney *U* test and Kruskal–Wallis test. A *p* value of < 0.05 was considered statistically significant.

## Results

**Fig. 1:**
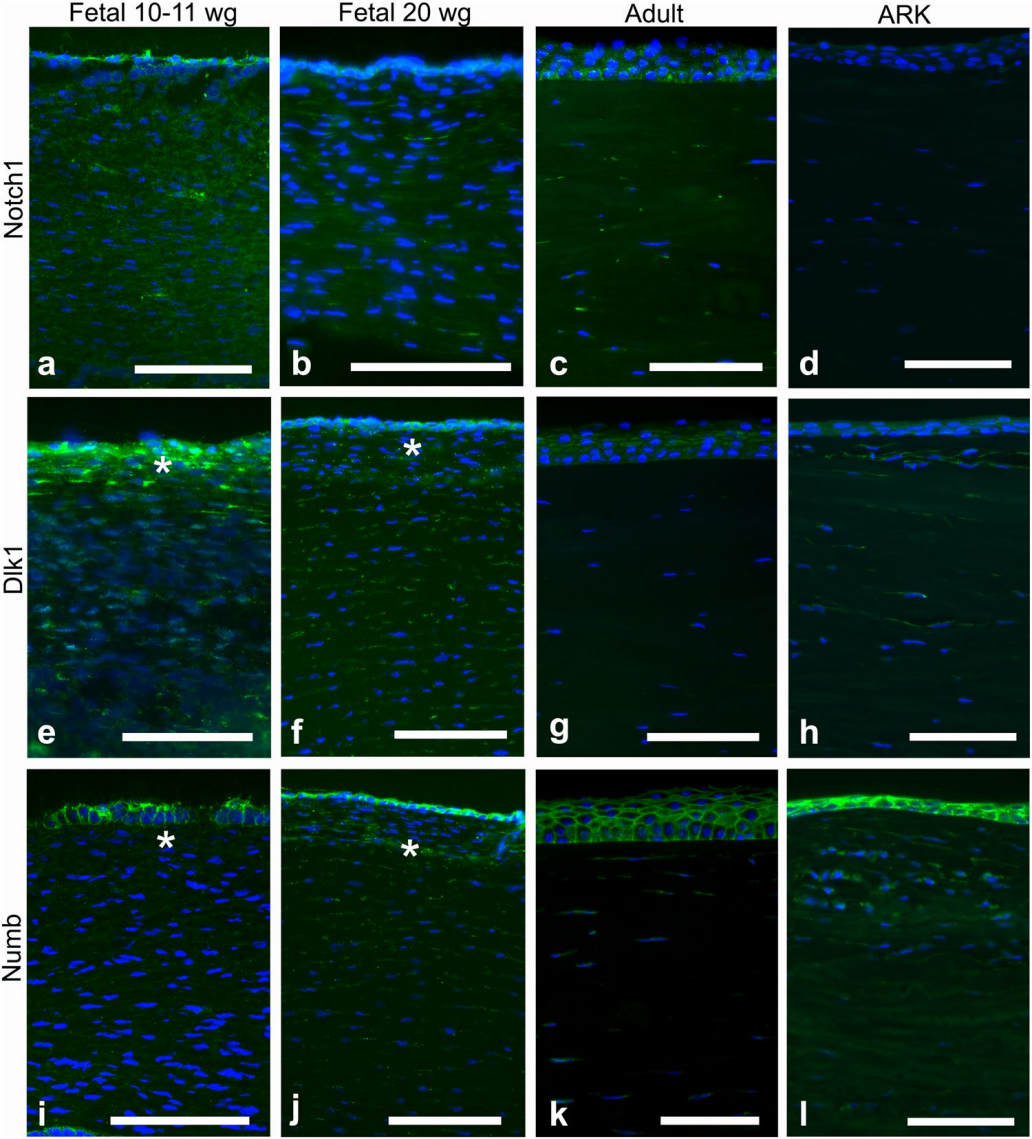
Cross-sections of fetal corneas 10–11 wg (**a**, **e**, **i**) and 20 wg (**b**, **f**, **j**) as well as normal adult corneas (**c**, **g**, **k**) and ARK (**d**, **h**, **l**) labeled with antibodies (green) against Notch1 (**a**–**d**), Dlk1 (**e**–**h**), and Numb (**i**–**l**). The corneal epithelium is shown at the top and the stroma below, in all photographs of all figures. Cell nuclei are labeled blue with DAPI (**a**–**l**). Immunolabeling against Notch1 (**a**–**d**) was detected as streaks in the stroma and strongly around the basal layers of the epithelium in all fetal and adult corneas (**a**–**c**), whereas in the ARK corneas, Notch1 was absent in the epithelium and only very scarce in the stroma (**d**). In the 20 wg fetal corneas, the labeling of the epithelium against Notch1 was slightly surpassed by the strong DAPI labeling of the epithelial cell nuclei (**b**). Dlk1 (**e**–**h**) labeled the epithelial cells and streaks in the stroma of all fetal corneas, more abundantly in the anterior region (**e**, **f**, asterisk), whereas in the adult corneas labeling was only present in the epithelium (**g**). In the ARK corneas, Dlk1 immunolabeling was present both in the epithelium and in streaks, more pronounced in the anterior pannus (**h**). In all fetal corneas, Abs against Numb (**i**–**l**), another inhibitor of Notch1, labeled the epithelial cells and streaks in the stroma (**i**, **j**, asterisk), but stromal labeling was more abundant in the 20 wg (**j**) than in the 10–11 wg fetal corneas (**i**). Numb labeling in adult corneas was present in the epithelial cells and in sporadic streaks in the stroma (**k**). In ARK, Numb immunolabeling was detected in the epithelium and anterior pannus (**l**) in a pattern similar to that of Dlk1. Bars, 100 μm

**Fig. 2:**
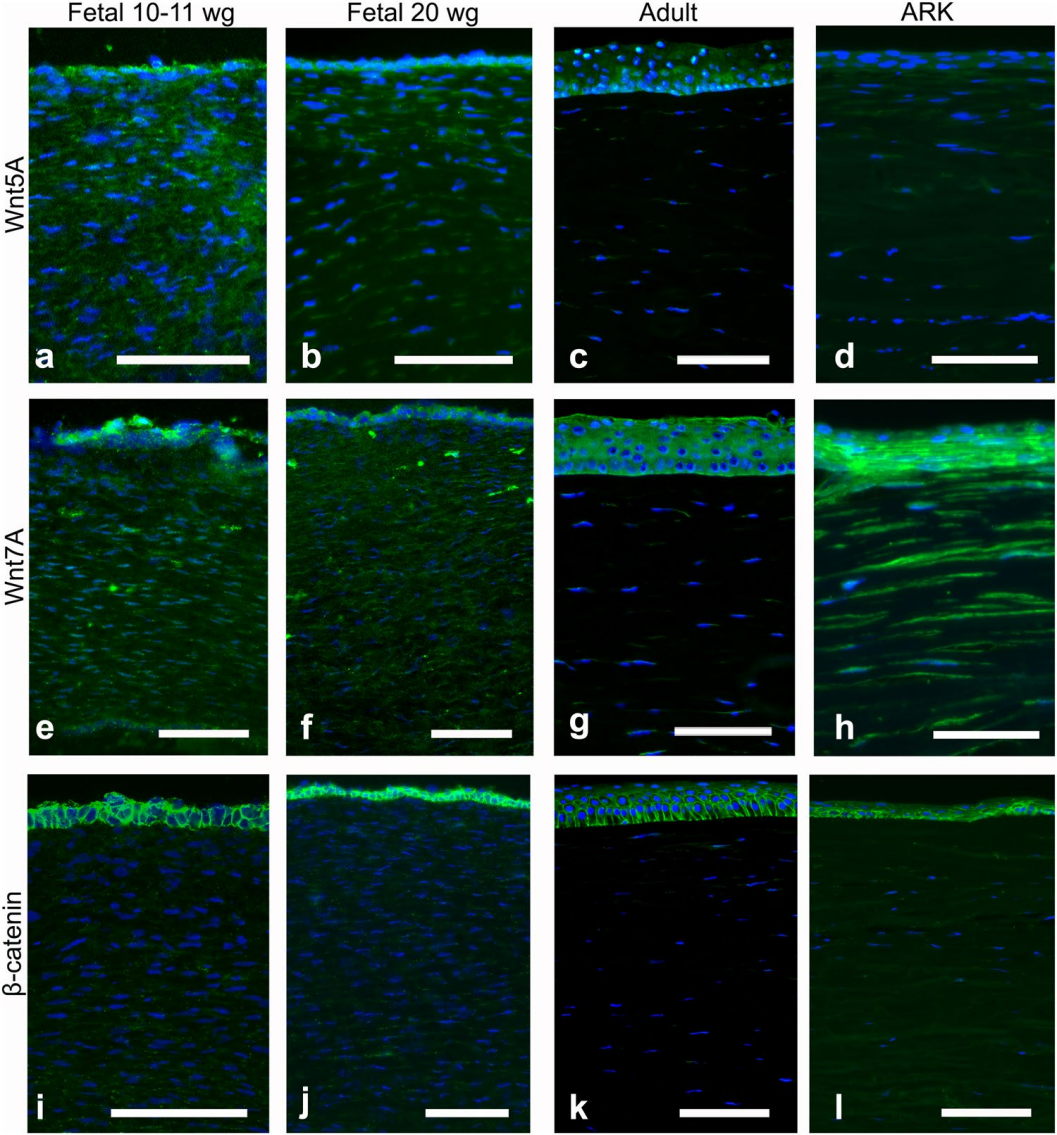
Cross-sections of fetal corneas 10–11 wg (**a**, **e**, **i**), 20 wg (**b**, **f**, **j**), normal adult corneas (**c**, **g**, **k**), and ARK (**d**, **h**, **l**) labeled with antibodies (green) against Wnt5a (**a**–**d**), Wnt7a (**e**–**h**), and β-catenin (**i**–**l**). Cell nuclei are labeled blue with DAPI (**a**–**l**). The Abs against Wnt5a abundantly labeled both the epithelial cells and streaks in the stroma of the fetal corneas (**a**, **b**). The stromal labeling was more abundant in the 10–11 wg (**a**) than in the 20 wg fetal corneas (**b**), in which the streaks were more profuse in the anterior region (**b**). In the adult corneas, only the epithelium was labeled (**c**), whereas in ARK corneas both the epithelium and the anterior pannus were labeled (**d**). Immunolabeling against Wnt7a was found in the epithelial cells and as stromal streaks in all fetal samples (**e**, **f**). In contrast, in the adult corneas, labeling was present in the epithelium but only in extremely sparse steaks in the stroma (**g**). In ARK, the epithelium and anterior pannus as well as the rest of the stroma were labeled by Wnt7a (**h**). β-Catenin immunolabeling was present abundantly in the contours of epithelial cells but only discretely in stromal streaks, in a similar pattern in the 10–11 wg (**i**) and 20 wg fetal corneas (**j**). In the adult corneas, labeling was present in the epithelial cells more intensively in the basal region but absent in the stroma (**k**). The staining pattern in the ARK corneas was similar to that of the fetal corneas, with β-catenin immunolabeling delineating the contours of epithelial cells and present in streaks in the anterior pannus (**l**). Bars, 100 μm

Although the epithelium was not optimally preserved in the fetal corneas, it was still possible to evaluate the staining patterns in all samples. The fetal corneas from 10–11 wg and 20 wg and the adult corneas presented similar patterns of immunolabeling with the antibody against Notch1 (Fig. 1a–c), seen as streaks (i.e., a patchy staining pattern) in the stroma and stronger staining around the basal layers of the corneal epithelium; however, the 10–11 wg fetal cornea showed increased presence of Notch1. In the ARK corneas, Notch1 was absent in the epithelium and only very scarce in the stroma (Fig. 1d). Dlk1 (Fig. 1e–f) labeled the epithelial cells and streaks in the stroma of all fetal corneas, more abundantly in the anterior region (Fig. 1f, asterisk), whereas in the adult corneas labeling was only present in the epithelium (Fig. 1g). In the ARK corneas, Dlk1 immunolabeling was present in the epithelium and in streaks in the anterior pannus (Fig. 1h). Numb labeling was observed in the epithelial cells of all fetal corneas, clearly delineating their contours, and as streaks in the stroma of all fetal corneas (Fig. 1i–j). Numb was, similarly to Dlk1, more abundant in the anterior stroma, particularly at 20 wg (Fig. 1j, asterisk). Conversely, Numb was only scarcely present in the stroma of adult corneas but was present in the epithelial cells (Fig. 1k). Numb immunolabeling was detected in the epithelium and anterior pannus of ARK corneas, in a pattern similar to that of Dlk1 (Fig. 1l). The epithelial cells of all fetal corneas were labeled with the Abs against Wnt5a (Fig. 2a–b), and the stroma was immunolabeled with these Abs in streaks, more abundantly in the 10–11 wg (Fig. 2a) than in the 20 wg fetal corneas (Fig. 2b). In the 20 wg fetal corneas, the immunolabeled streaks were slightly more abundant in the anterior stroma (Fig. 2b). In contrast, only the epithelium was labeled with the Abs against Wnt5a in the adult corneas and the stroma was unlabeled (Fig. 2c), whereas in ARK corneas both the epithelium and the anterior pannus were labeled (Fig. 2d). Immunolabeling with Abs against Wnt7a was identified in the epithelial cells and as streaks in the stroma of all fetal corneas (Fig. 2e–f). Labeling for Wnt7a in the stroma of all fetal corneas presented as streaks that, similarly to Wnt5a, were slightly more abundant in the 10–11 wg (Fig. 2e) than in 20 wg corneas (Fig. 2f). The Abs against Wnt7a labeled the epithelium in adult corneas, but in the stroma only extremely sparse streaks were labeled (Fig. 2g). In the ARK corneas, Wnt7a immunolabeling was present in the epithelium and anterior pannus as well as in the remaining of the stroma (Fig. 2h). The Ab against β-catenin intensely labeled the contours of the epithelial cells in a similar pattern in both the 10–11 wg (Fig. 2i) and 20 wg (Fig. 2j) fetal corneas. In the stroma of all fetal corneas, this Ab labeled only discrete streaks (Fig. 2i–j). In the adult corneas, labeling with the Ab against β-catenin was completely absent in the stroma, but the epithelial cells were labeled in a pattern that was stronger in the basal region (Fig. 2k). The staining pattern in the ARK corneas was similar to that of the fetal corneas, with β-catenin immunolabeling delineating the contours of epithelial cells and present in streaks in the anterior pannus (Fig. 2l).

**Fig. 3:**
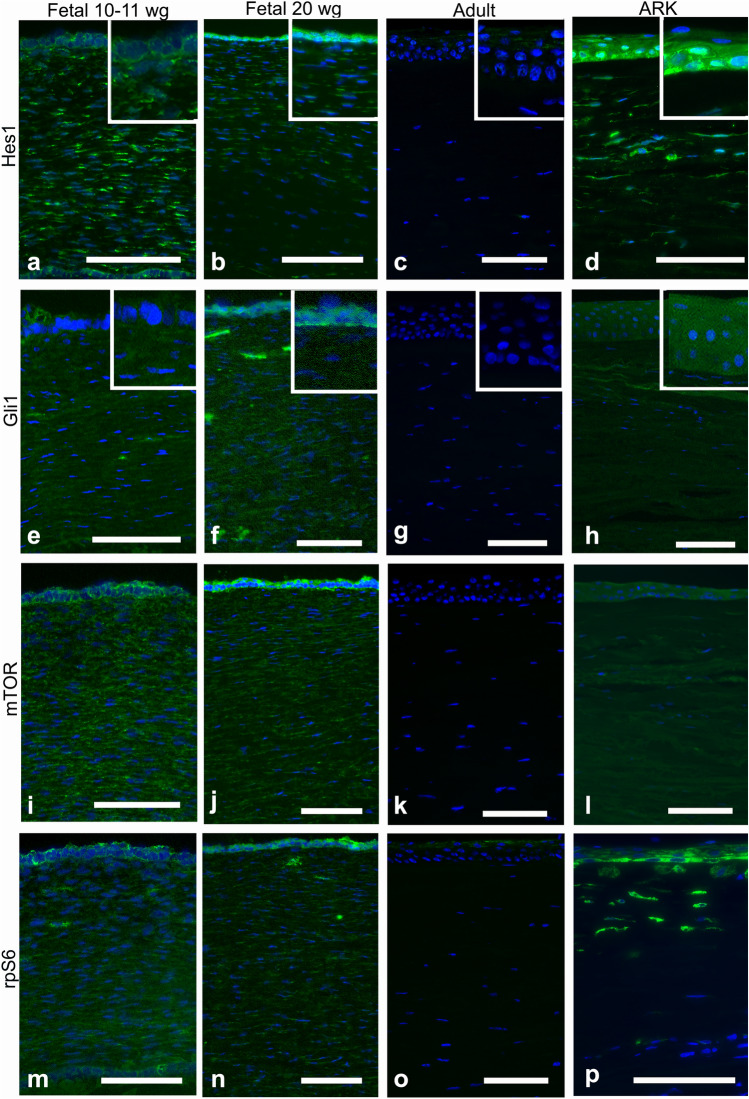
Cross-sections of fetal corneas 10–11 wg (**a**, **e**, **i**, **m**) and 20 wg (**b**, **f**, **j**, **n**), adult normal corneas (**c**, **g**, **k**, **o**), and ARK (**d**, **h**, **l**, **p**) labeled with antibodies (green) against Hes1 (**a**–**d**), Gli1 (**e**–**h**), mTOR (**i**–**l**), and p-rpS6 (**m**–**p**). Tissue preservation of the fetal tissue was variable. Cell nuclei are labeled blue with DAPI (**a**–**p**). Abs against Hes1 strongly immunolabeled the epithelial cells of the fetal corneas (**a**, **b**). These Abs labeled streaks in the stroma more profusely in the 10–11 wg (**a**) than in the 20 wg fetal corneas (**b**) and more intensively in the anterior stroma (**b**). In contrast, in the adult corneas, immunolabeling was not detected (**c**), whereas Hes1 was present in the epithelium and in the anterior stroma of the ARK corneas (**d**). Immunolabeling with Abs against Gli1 was present in the epithelial cells in all fetal corneas (**e**, **f**). Labeling in the stroma was present in streaks in all fetal corneas (**e**, **f**). Immunolabeling for these Abs was not observed in adult corneas (**g**). In the ARK corneas, the Abs against Gli1 labeled the epithelium and the anterior pannus (**h**). The inserts in **a**–**h** show the epithelium at higher magnification. The Ab against mTOR abundantly labeled the epithelial cells of all fetal corneas (**i**, **j**) but marked the epithelium in the 20 wg fetal corneas more intensively (**j**). The stroma in all fetal corneas was labeled in streaks (**i**, **j**) but was more abundantly marked in the 10–11 wg fetal corneas (**i**). This Ab did not immunolabel the adult corneas (**k**), but the epithelium and anterior pannus were labeled by this Ab in the ARK corneas (**l**). The Ab against p-rpS6 labeled the epithelial cells and abundant streaks in the stroma of all fetal corneas in a likewise pattern in both 10–11 wg (**m**) and 20 wg fetal corneas (**n**). The stroma in the adult corneas was not labeled and the surface of the epithelium was only scarcely labeled, suggesting sticky adherence to the epithelial surface (**o**). In the ARK corneas, immunostaining against p-rpS6 was present in the epithelium and anterior pannus (**p**). Bars, 100 μm

The Abs against Hes1 strongly labeled the epithelium of all fetal corneas (Fig. 3a–b). These Abs labeled the stroma in streaks, more abundantly in the 10–11 wg (Fig. 3a) than in the 20 wg fetal corneas (Fig. 3b), and in the latter immunolabeling was more marked in the anterior stroma (Fig. 3b). These Abs did not label the epithelium or the stroma in the adult corneas (Fig. 3c), whereas they labeled the epithelium and the anterior stroma in the ARK corneas (Fig. 3d). The epithelial cells in all fetal corneas were labeled with the Abs against Gli1 (Fig. 3e–f), and the labeling was more abundant in the basal region of the epithelium in the 20 wg fetal corneas (Fig. 3f). Immunolabeling with these Abs was found in streaks in the stroma of all fetal corneas (Fig. 3e–f) and was stronger at 20 wg (Fig. 3f), whereas it was completely absent in the epithelium and stroma of adult corneas (Fig. 3g). In the ARK corneas, the Abs against Gli1 labeled the epithelium and the anterior pannus (Fig. 3h).

The Ab against mTOR labeled the epithelial cells in all fetal corneas (Fig. 3i–j) but more strongly in the 20 wg fetal corneas (Fig. 3j). The stroma of all fetal corneas presented labeled streaks (Fig. 3i–j), which were more abundant in the 10–11 wg fetal corneas (Fig. 3i), whereas in the adult corneas no immunolabeling against mTOR was detected (Fig. 3k). In contrast, the epithelium and anterior pannus were labeled by this Ab in the ARK corneas (Fig. 3l). Immunostaining against p-rpS6 was present in the epithelial cells and in abundant streaks in the stroma of all fetal corneas, with similar intensity in both 10–11 wg and 20 wg (Fig. 3m–n). In the adult corneas, labeling with the Ab against p-rpS6 was absent in the stroma and it was only barely present in the surface of the epithelium, which could be indicative of nonspecific adherence to the epithelial surface (Fig. 3o). In the ARK corneas, immunostaining against p-rpS6 was present in the epithelium and anterior pannus (Fig. 3p).

**Fig. 4:**
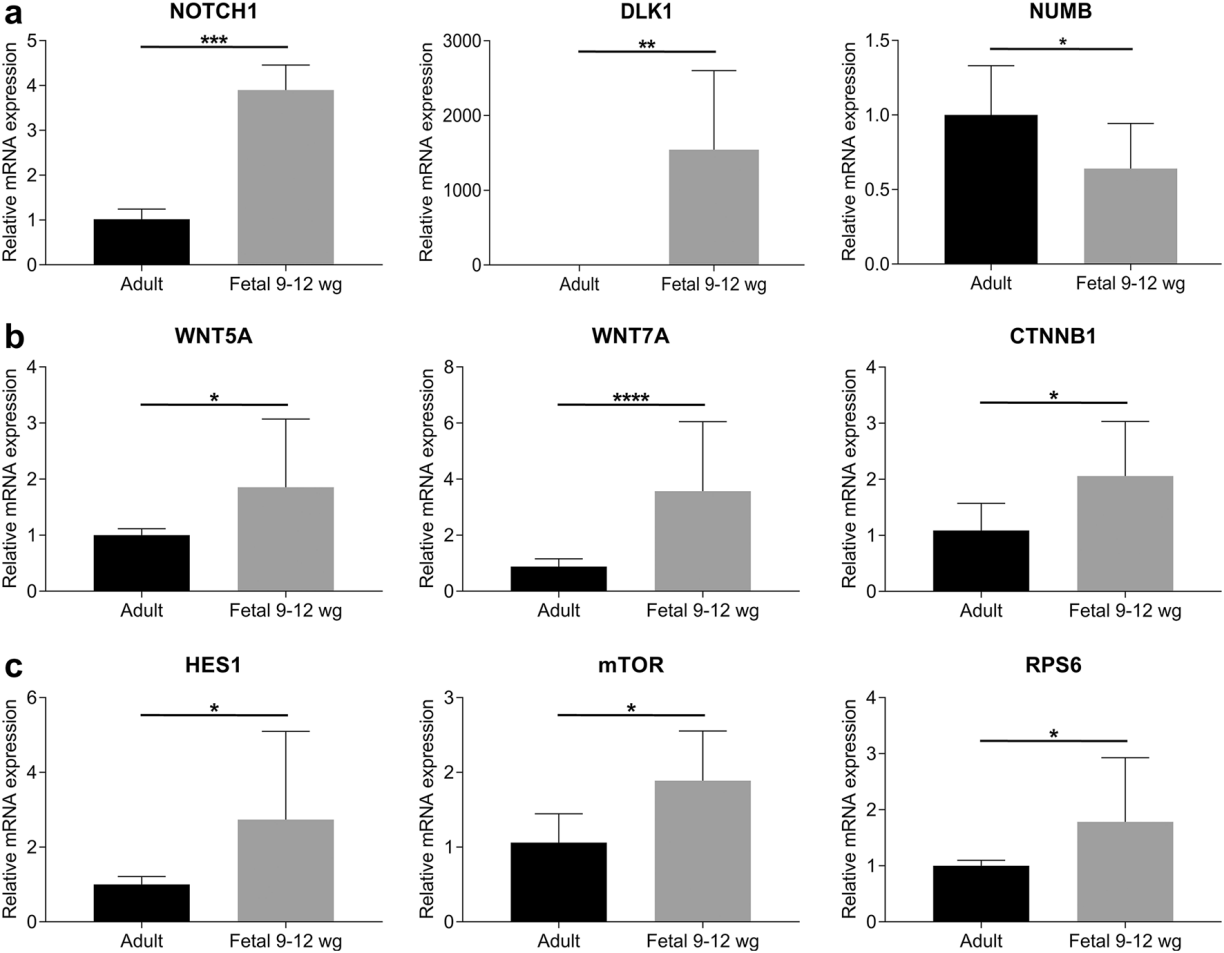
Gene expression of Notch1 (*NOTCH1*), Dlk1 (*DLK1*), Numb (*NUMB*) (**a**), Wnt5A (*WNT5A*), Wnt7A (*WNT7A*), β-catenin (*CTNNB1*) (**b**), and Hes1 (*HES1*), mTOR (*mTOR*), and rps6 (*RPS6*) (**c**) in 9–12 wg fetal corneas as compared with adult cornea. There was a 3.89-fold increase in *Notch1* gene expression, 1540-fold increase in *Dlk1* gene expression, and 0.64-fold decrease in *Numb* gene expression in 9–12 wg fetal corneas. (**a**). Expression of *Wnt5A*, *Wnt7A*, and *β-catenin *genes was increased by 1.85-, 3.57-, and 2.05-fold, respectively. (**b**). Gene expression of *Hes1*, *mTOR*, and *rps6* was increased by 2.73-, 1.89-, and 1.78-fold, respectively (**c**). Values are mean ± SD. *p < 0.05; **p < 0.01; ***p < 0.001; ****p < 0.0001

We subsequently examined the gene expression profiles of the cell signaling pathways by RT-qPCR. The central part of the 9 wg, 10–11 wg fetal cornea, 12 wg fetal cornea (pooled together; 9–12 wg), and three adult corneas was collected from frozen tissue sections using laser microdissection microscopy. Compared with the adult corneas, expression of *Notch1* gene was 3.89-fold higher in 9–12 wg fetal corneas. We also found that gene expression of *Dlk1* was increased by 1540-fold. However, gene expression of *Numb* was decreased by 0.64-fold (Fig. 4a). Expression of *Wnt5A, Wnt7A*, and *β-catenin* genes was increased by 1.85-, 3.57-, and 2.05-fold, respectively (Fig. 4b). Gene expression of *Hes1*, *mTOR*, and *rps6* was increased by 2.73-, 1.89-, and 1.78-fold respectively (Fig. 4c). Additionally, we investigated whether the age of the fetus played a role in gene expression. We compared the fold gene expression between 9 and 12 wg fetal corneas. We found that significant differences in gene expression between 9 and 12 wg fetal corneas were found for *Hes1* and *rps6*. *Notch1*, *Dlk1*, *Numb*, *Wnt5A*, *Wnt7A*, *β-catenin*, and *mTOR* gene expression was not significantly different between 9 and 12 wg fetal corneas (Supplemental Fig. 1).

## Discussion

The present study is the first to investigate the patterns of immunolabeling and gene expression of key cell signaling pathways related to cell proliferation and differentiation (Notch1, Wnt/β-catenin, SHH, and mTOR) in the human fetal cornea. Cell differentiation is altered in ARK (Latta et al. 2018; Ramaesh et al. 2005), and we have previously described changes in these cell signaling pathways in naïve aniridia corneas and in failed corneal transplants with advanced ARK (Vicente et al. 2018b). The present study allows a comparison between human fetal, normal adult, and ARK corneas, addressing the question of whether there is an immature host milieu in ARK that mimics that encountered during fetal maturation.

The main limitation of this study is the reduced number of human corneal fetal and ARK samples derived from the scarcity of opportunity to collect such samples. On the other hand, the fact that the data were collected on human samples is a major strength of this study. We confirm previous results regarding Notch1 and its negative regulators (Dlk1 and Numb), in the epithelium and stromal pannus of ARK corneas (Vicente et al. 2018b) compared with normal adult corneas. Notch1 is responsible for corneal epithelial cell differentiation (Ma et al. 2007), and lack of Notch1 in a mouse model leads to keratinized hyperplastic, skin-like corneal epithelium with neovascularization in the stroma (Vauclair et al. 2007), which reproduces, in part, the changes encountered in the ARK corneas. In contrast, in fetal corneas, immunolabeling with Notch1 was detected in a similar pattern to that of normal adult corneas, with 10–11 wg fetal corneas showing slightly increased presence of Notch1. *Notch1* gene expression analysis revealed that it is expressed more abundantly in the fetal corneas. Nevertheless, the negative regulators of Notch1, Dlk1 and Numb, were also present in the stroma of the fetal corneas. This cell signaling pathway has a very diverse role in development and in self-renewing tissues, such as the corneal epithelium, having functions that range from serving as a gatekeeper for progenitor cells to playing a role in cell lineage differentiation and barrier function regulation (Ma et al. 2007), and we have now described a stronger detection of the key components of the Notch1 pathway in human fetal corneas during development. Dlk1 helps cells remain immature, secures progenitor cell populations (Abdallah et al. 2004), and is important in fetal development. Here, we have revealed that it was abundantly expressed in the epithelial cells and stroma of fetal corneas in a similar pattern to that of ARK corneas. There is a lack of data on the possible roles of Numb and Dlk1 in the corneal stroma. Numb has been shown to contribute to the maintenance of an undifferentiated state in epithelial progenitor cells in epidermis (Iannolo et al. 2016), and the present findings can be interpreted as indicating the possibility of a similar role in the human cornea.

The Wnt/β-catenin cell signaling pathway has a decisive role in determining cell fate, proliferation, differentiation, and apoptosis during development, as well as maintenance of stem cells and homeostasis in adult tissues (Logan and Nusse 2004), and its presence was enhanced in the epithelium and pannus of corneas with advanced ARK, confirming our previous report (Vicente et al. 2018b). Upregulation of the Wnt5a and Wnt7a signaling pathway in fetal corneas was similar to what has been described in other ocular tissues during eye development (Borello et al. 1999; Ang et al. 2004). A previous study in mice established that conditional ablation of β-catenin led to precocious stratification of the corneal epithelium, postulating that this signaling pathway plays a crucial role in corneal morphogenesis (Li et al. 2015). Furthermore, it has been shown that the Wnt/β-catenin cell signaling pathway influences the development of different ocular cell types in fetal corneas, including epithelial limbal stem cells, epithelial cells (Han et al. 2014; Lee et al. 2017), and keratocytes (Li et al. 2015). In addition, it has been demonstrated in a mouse model that Wnt/β-catenin cell signaling is not present in adult corneal limbus but that it is active in wing and squamous corneal epithelium and in developing corneal stroma and endothelium, in contrast to what occurs in adult stroma and endothelium (Wang et al. 2018). Here we have shown that the staining patterns regarding the Wnt/β-catenin signaling pathway in the epithelium and stroma of human fetal corneas seem to mimic a pattern of activation of this signaling pathway described previously in mice (Li et al. 2015). This signaling pathway seems to have a complex and multifaceted role in the development and maintenance of the corneal epithelial cells (Wang et al. 2018).

SHH is a secreted protein that works as a morphogen during development (McMahon 2000) and directly regulates the cell cycle, promoting proliferation by upregulating cyclin D1 (Oliver et al. 2003). Here we found a stronger detection of elements of the SHH signaling pathway (Gli1 and Hes1) in 10–11 wg and 20 wg corneas, as well as increased Hes1 gene expression in 9–12 wg fetal corneas, suggesting that activation of this pathway might be important during fetal corneal development. Hes1 has a role in preventing differentiation and maintaining progenitor cells and proliferation during development (Lee et al. 2005). Its abundant detection in the epithelial cells and stroma of fetal corneas, compared with its absence in adult corneas, suggests a role in cell proliferation during development and mimics the pattern found in ARK corneas. SHH is upregulated in migrating corneal epithelial cells (Saika et al. 2004). Human mutations in SHH result in holoprosencephaly, which includes anophthalmia and cyclopia (Belloni et al. 1996; Roessler et al. 1996). Furthermore, it has been proven that loss of SHH signaling in the lens disturbs the migration of neural crest cells into the cornea (Choi et al. 2014). Therefore, SHH signaling has both direct and indirect effects on corneal development (Choi et al. 2014). The results in the present study further emphasized its importance during corneal fetal development and the important parallel with the patterns encountered in ARK corneas.

The mTOR cell signaling pathway can be described as a central regulator of cell proliferation, growth, motility, transcription, protein synthesis, autophagy, and survival (Dowling et al. 2010; Hagglund et al. 2017). It regulates apoptosis and cell proliferation in pterygium (Liu et al. 2017) and was suggested to be upregulated in the corneal epithelium and pannus in advanced ARK (Vicente et al. 2018b). In the present study, both mTOR and p-rpS6, a downstream element of this cell signaling pathway, were found in the fetal corneal epithelial cells and stroma but were absent in normal adult corneas, suggesting that this signaling pathway is also upregulated in fetal corneas at 10–11 wg and 20 wg, where cell proliferation is most likely central. The gene expression analysis also showed that mTOR and rps6 expression were higher in fetal corneas. The contribution of this signaling pathway to proliferation has led to studies where its inhibition was used to treat corneal neovascularization (Shin et al. 2013) and transplant rejection (Zhu et al. 2014). The effects of rapamycin, an inhibitor of the mTOR signaling pathway, in human corneal epithelial cells in vitro have been previously tested, indicating that it prevents the loss of corneal epithelial stem cells to replicative senescence and apoptosis (Gidfar et al. 2017). Nevertheless, the effects of this signaling pathway on corneal development are not known and should be further explored.

Our findings related to the immunolabeling pattern for these cell signaling pathways in the basal layers of the corneal epithelium in fetal corneas, with labeling of areas usually populated by TAC in adults (Zhou et al. 2006), raised attention to the importance of epithelial basal cells with high migratory and proliferative capacity in normal adults and ARK corneas. These cells are formed by amplification of epithelial limbal stem cells and migrate centripetally during normal corneal epithelial homeostasis and wound healing in response to growth factors, cytokines, and changes in the extracellular matrix (Lehrer et al. 1998; Zhou et al. 2006). The importance of these processes and TAC in chronic wound healing in ARK remains a question to be further explored, but there is an apparent similarity between the regulation of cell signaling pathways related to the activation of TAC in normal adults, epithelium in ARK corneas and in high proliferative fetal epithelial cells.

The *PAX6 *gene is known to regulate transduction in ocular cells and to have a crucial role in securing human corneal epithelium identity by regulating cell differentiation (Kitazawa et al. 2017). Here we also describe how Notch1, Wnt/β-catenin, SHH, and mTOR cell signaling pathways are altered in patients with ARK, a condition resulting from reduced Pax6 protein levels. With the exception of Notch1, the changes in the cell signaling pathways observed in the corneas of patients with ARK and the patterns detected on human fetal corneas at 9–12 wg, 10–11 wg, and 20 wg were analogous. This suggests that, in corneas with advanced ARK, with reduced Pax6 protein levels, the cellular microenvironment mimics a less differentiated milieu, similar to that occurring during normal fetal development in corneas with normal Pax6 protein levels.

In this study, we analyzed localization and quantified the expression of Notch1, Wnt/β-catenin, SHH, and mTOR in normal human fetal corneas and healthy adult corneas. Cell signaling in the human cornea during development is currently poorly understood, but our data have highlighted that certain pathways including mTOR1 and SHH may be essential in differentiating epithelial cells. Similarity to what is present during normal corneal fetal development, a more undifferentiated milieu, supports the proposed importance of host-specific factors and the corneal microenvironment in the context of limbal stem cell deficiency in ARK. We found that there are substantial similarities, excluding activation of *Notch1*, between the gene expression profiles of key signaling components between fetal and ARK corneas.

## Supplementary Information

Below is the link to the electronic supplementary material.

Supplementary file1 Gene expression of Notch1 (*NOTCH1*), Dlk1 (*DLK1*), and Numb (*NUMB*) (a), Wnt5A (*WNT5A*), Wnt7A (*WNT7A*), and β-catenin (*CTNNB1*) (b), and Hes1 (*HES1*), mTOR (*mTOR*), and rps6 (*RPS6*) (c) in 9 wg and 12 wg fetal corneas as compared with adult cornea. Significant differences in fold change gene expression between 9 wg and 12 wg fetal corneas were found for Hes1 and rps6. Notch1, Dlk1, Numb, Wnt5A, Wnt7A, β-catenin, and mTOR gene expression was not significantly different between 9 wg and 12 wg fetal corneas. Values are mean ± SD. *n.s*. not significant, *p<0.05; **p<0.01; ***p<0.001. (TIF 909 KB)

## Data Availability

The data that support the findings of this study are available on request from the corresponding author. The data are not publicly available owing to privacy or ethical restrictions.
