# Supplementary figures and images for: Aniridia-related keratopathy relevant cell signaling pathways in human fetal corneas
Source: Histochem Cell Biol. 2022 May 12;158(2):169–80. doi: 10.1007/s00418-022-02099-9 (PMC9338123; doi:10.1007/s00418-022-02099-9)

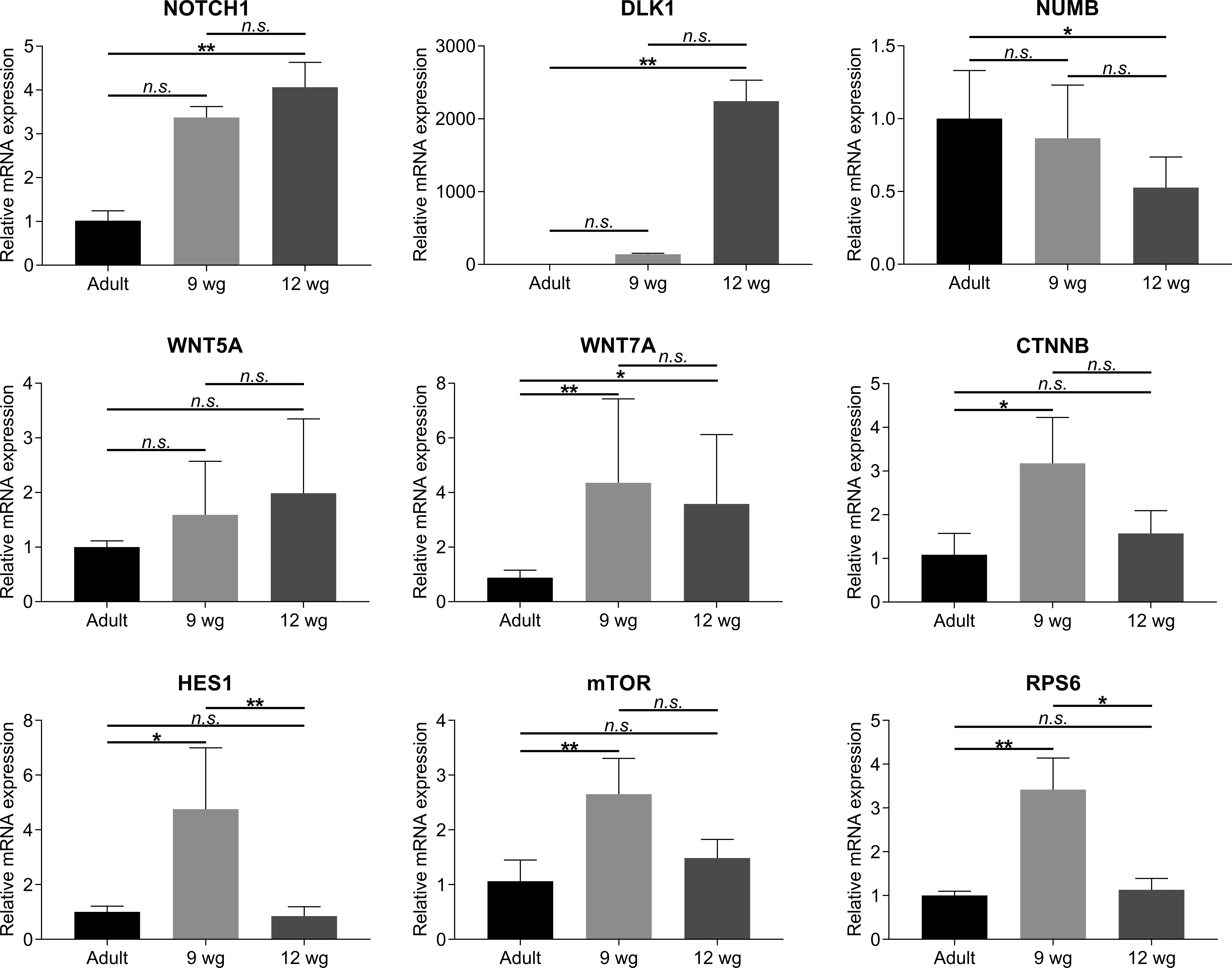

Supplement: Supplementary file 1 — Supplementary file1 Gene expression of Notch1 (NOTCH1), Dlk1 (DLK1), and Numb (NUMB) (a), Wnt5A (WNT5A), Wnt7A (WNT7A), and β-catenin (CTNNB1) (b), and Hes1 (HES1), mTOR (mTOR), and rps6 (RPS6) (c) in 9 wg and 12 wg fetal corneas as compared with adult cornea. Significant differences in fold change gene expression between 9 wg and 12 wg fetal corneas were found for Hes1 and rps6. Notch1, Dlk1, Numb, Wnt5A, Wnt7A, β-catenin, and mTOR gene expression was not significantly different between 9 wg and 12 wg fetal corneas. Values are mean ± SD. n.s. not significant, *p<0.05; **p<0.01; ***p<0.001. (TIF 909 KB) [file 418_2022_2099_MOESM1_ESM.tif]
